# SHP-2 phosphatase contributes to KRAS-driven intestinal oncogenesis but prevents colitis-associated cancer development

**DOI:** 10.18632/oncotarget.11601

**Published:** 2016-08-25

**Authors:** Jessica Gagné-Sansfaçon, Geneviève Coulombe, Marie-Josée Langlois, Ariane Langlois, Marilene Paquet, Julie Carrier, Gen-Sheng Feng, Cheng-Kui Qu, Nathalie Rivard

**Affiliations:** ^1^ Department of Anatomy and Cell Biology, Cancer Research Pavilion, Faculty of Medicine and Health Sciences, Université de Sherbrooke, Sherbrooke, QC, Canada; ^2^ Département de microbiologie et pathologie, Université de Montréal, St-Hyacinthe, QC, Canada; ^3^ Department of Medicine, Université de Sherbrooke, Sherbrooke, QC, Canada; ^4^ Department of Pathology and Division of Biological Sciences, University of California San Diego, La Jolla, CA, USA; ^5^ Department of Pediatrics, Emory University School of Medicine, Atlanta, GA, USA

**Keywords:** SHP-2, colorectal cancer, colitis-associated cancer, oncogene, mitogen-activated protein kinase

## Abstract

A major risk factor of developing colorectal cancer (CRC) is the presence of chronic inflammation in the colon. In order to understand how inflammation contributes to CRC development, the present study focused on SHP-2, a tyrosine phosphatase encoded by *PTPN11* gene in which polymorphisms have been shown to be markers of colitis susceptibility. Conversely, gain-of-function mutations in *PTPN11* gene (E76 residue) have been found in certain sporadic CRC. Results shown herein demonstrate that SHP-2 expression was markedly increased in sporadic human adenomas but not in advanced colorectal tumors. SHP-2 silencing inhibited proliferative, invasive and tumoral properties of both intestinal epithelial cells (IECs) transformed by oncogenic KRAS and of human CRC cells. IEC-specific expression of a SHP-2^E76K^ activated mutant in mice was not sufficient to induce tumorigenesis but markedly promoted tumor growth under the *Apc^Min/+^* background. Conversely, mice with a conditional deletion of SHP-2 in IECs developed colitis-associated adenocarcinomas with age, associated with sustained activation of Wnt/β-catenin, NFκB and STAT3 signalings in the colonic mucosae. Moreover, SHP-2 epithelial deficiency considerably increased tumor load in *Apc^Min/+^* mice, shifting tumor incidence toward the colon. Overall, these results reveal that SHP-2 can exert opposing functions in the large intestine: it can promote or inhibit tumorigenesis depending of the inflammatory context.

## INTRODUCTION

The pathogenic mechanisms driving colorectal cancer (CRC) development are complex and heterogeneous. A major component increasing the risk of CRC is the presence of inflammatory bowel diseases (IBD), particularly ulcerative colitis (UC) [1–2]. For both UC-associated CRCs and sporadic CRCs, many mutations occur during the carcinogenic process. The development of sporadic CRC generally involves alterations in the oncogene *KRAS* and the tumor suppressor genes *APC*, *SMAD4* and *TP53* [3–5]. The pathogenesis of UC-associated CRC appears to differ, involving an orderly progression from inflamed and hyperplastic epithelia to flat dysplasia and lastly to adenocarcinoma. The increased risk to develop CRC in UC patients is presumably attributable to the long-term harmful effects of sustained inflammation in the colon of these patients [1]. One deleterious effect of chronic inflammation is the increased production of reactive oxygen species (ROS) causing oxidative DNA damage [5]. Loss of expression or mutation of the tumor-suppressor gene *TP53* is probably a key early event in UC-associated carcinogenesis [1–5]. Nevertheless, our knowledge of the underlying cellular mechanisms involved in this process remains incomplete.

Notably, polymorphisms in the *PTPN11* gene were previously associated with increased susceptibility to develop UC [6]. *PTPN11* gene codes for the Src homology 2-domain containing tyrosine phosphatase (SHP-2) which is ubiquitously expressed and which closely regulates several cell processes including proliferation, differentiation, chemotaxis and survival [7]. Genetic and biochemical evidence demonstrate that SHP-2 positively regulates the RAS/Mitogen-Activated Protein Kinase (MAPK) pathway activation by most receptors [7–10]. SHP-2 binds directly to selected tyrosine kinase receptors or, more often, to scaffolds and becomes activated. Actions of SHP-2 on JAK/STATs (1,3,5) [11], NFκB [9], PI3K/AKT [12] and RHOA [13] pathways were also reported in various cellular contexts.

To characterize the functional role of SHP-2 in intestinal homeostasis, we have recently generated mice with a deletion of Shp-2 expression specifically in intestinal epithelial cells (IECs). Importantly, these mice (*Shp-2*^IEC-KO^) develop spontaneously inflammation in their colon [14]. Chemokine and cytokine secretion profiles indicate that the immunophenotype of *Shp-2*^IEC-KO^ mice is similar to patients suffering from UC as opposed to Crohn's disease. Additionally, *SHP-2* gene transcripts are reduced in intestinal biopsies from patients with active UC, indicating an inverse relationship between SHP-2 expression and intestinal inflammatory phenotype [14]. Of note, the activity of pro-inflammatory transcription factors STAT3 and NFκB are markedly enhanced in SHP-2-deficient colonocytes [14]. Likewise, mice with hepatocyte-specific deletion of *Shp-2* exhibited excessive STAT3 activation and developed severe liver inflammation and tumors with age [15]. Importantly, sustained activation of STAT3 and NFκB are critical for the development of colitis-associated cancer [1,16–17]. Whether loss of epithelial SHP-2 represents an initiating event in colorectal tumorigenesis in the context of chronic inflammation remains however to be determined.

Paradoxically, in humans, gain-of-function mutations of *PTPN11* gene have been associated with pediatric leukemias and certain solid carcinomas including hepatocellular carcinoma and CRC [18–19]. These specific mutations increase SHP-2 phosphatase activity and enhance its binding to signaling partners resulting in sustained activation of downstream effectors, particularly the RAS/MAPK pathway [19–20]. Importantly, dysregulation of this pathway is also a common event in sporadic colorectal carcinogenesis. Indeed, activating mutations in *KRAS*, *NRAS* and *BRAF* genes are found in up to 60% of CRCs and are acquired at an early premalignant stage consistent with a role in tumor initiation and/or progression [21–22].

Based on these results, we speculate that SHP-2 can function as an oncoprotein (through the overactivation of the RAS/MAPK pathway) but that under an inflammatory context, it can also act as a tumor suppressor. The present study was therefore designed to elucidate the significance of epithelial SHP-2 in colorectal tumorigenesis.

## RESULTS

### SHP-2 expression is increased in early stage colorectal tumors

**Figure 1:**
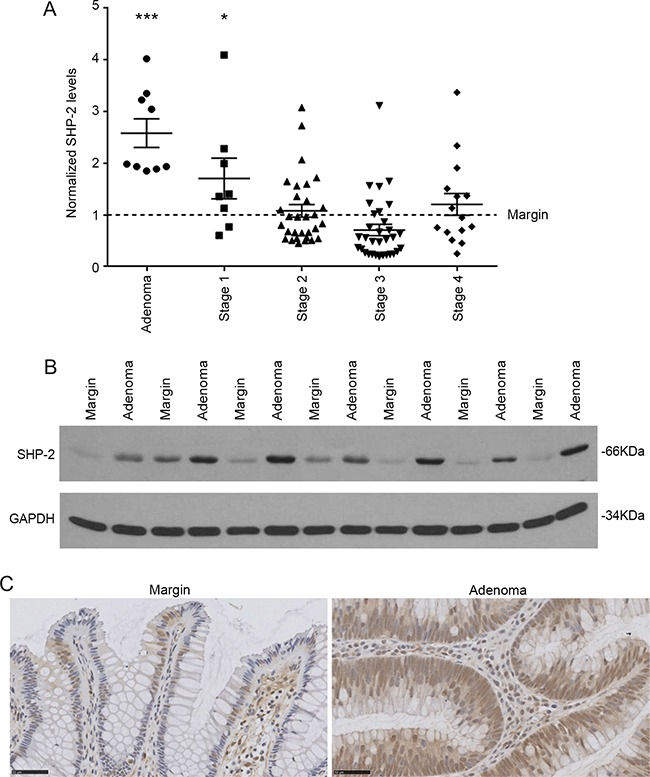
SHP-2 expression in sporadic human colorectal tumors **A.** Relative *SHP-2* mRNA levels were determined by q-PCR in human advanced adenomas and adenocarcinomas compared to the paired adjacent healthy tissue (Adenoma N=9; Stage 1 N=8; Stage 2 N=30; Stage 3 N=32; Stage 4 N=15; paired Student's *t*-test). Relative expression was normalized with the housekeeping genes *MRPL19, SDHA* and *YWHAZ.* **B.** Representative immunoblot analysis of SHP-2 protein performed on protein extracts from paired resection margins and adenoma specimens. GAPDH expression is shown as a protein loading control. **C.** Representative SHP-2 immunohistochemistry performed on human adenomas compared to paired adjacent healthy tissue. Scale bars: 50μM. Data are expressed as mean ± SEM. *p≤0.05, ***p≤0.001.

SHP-2 mRNA levels were first examined in sporadic human colorectal advanced adenomas and adenocarcinomas at various stages. As illustrated in Figure 1A, transcript levels of *SHP-2* were significantly enhanced in colorectal adenomas and stage 1 tumors but not in more advanced stages. SHP-2 protein expression was further analyzed by Western blot to verify if the increased SHP-2 mRNA levels observed in adenomas could be correlated with enhanced protein levels. As shown in Figure 1B, SHP-2 protein levels were also increased in all analyzed human colorectal adenomas compared to normal adjacent tissues. Furthermore, immunohistochemistry analyses demonstrated that the increased expression of SHP-2 was primarily observed in the hyperplastic epithelium and not in the lamina propria (Figure 1C). Thus, these results suggest that increased transcription may contribute to a greater expression of SHP-2 protein in early stages of sporadic CRC.

### SHP-2 silencing inhibits proliferative, invasive and tumoral properties of IECs transformed by oncogenic KRAS and human CRC cells

**Figure 2:**
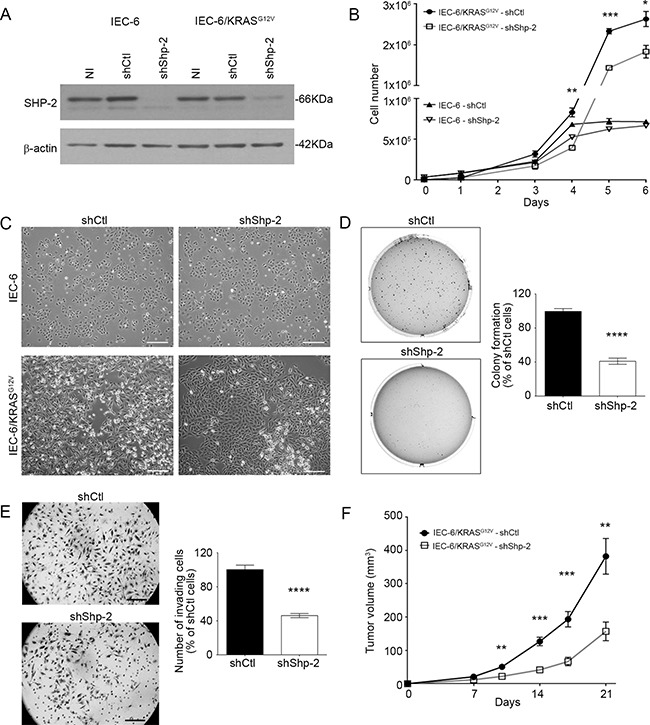
SHP-2 is required for oncogenic activity of KRAS^G12V^ in intestinal epithelial cells **A.** Control IEC-6 and KRAS^G12V^-expressing cells were stably infected with lentiviruses encoding for a control shRNA (scrambled sequence, shCtl) or encoding Shp-2-specific shRNA (shShp-2). After selection, stable cell populations were lysed and protein lysates were analyzed by Western blot for SHP-2 and β-actin protein expression. NI: *Non-infected. **B.** Cells were seeded in a 6-well plate, harvested and counted after different time intervals. Growth curves were compared between shCtl and shShp-2 for both control IEC-6 cells and transformed KRAS^G12V^ cells. **C.** Representative phase-contrast microscopy images of control IEC-6 and KRAS^G12V^ cells expressing shShp-2 or shCtl. Bars: 50 μm. **D.** IEC-6 KRAS^G12V^ cells stably expressing shShp-2 or shCtl were cultured in soft agarose for 10 days before 3-(4,5-Dimethylthiazol-2-Yl)-2,5-Diphenyltetrazolium Bromide (MTT) staining. **E.** Invasion capacity of IEC-6 KRAS^G12V^ cells stably expressing shShp-2 or shCtl was studied using Matrigel-coated Transwells after 48h. The number of cells was determined in 10 fields and the experiments performed in duplicate. **F.** Tumor growth over time was measured after subcutaneous injection of 5×10^5^ IEC-6 KRAS^G12V^ cells stably expressing shShp-2 or shCtl. Results represent mean tumor volume obtained from at least 13 mice injected for each cell line. (A-E) All experiments were conducted on at least three different cell populations (different infections). Data are expressed as mean ± SEM. *p≤0.05, **p≤0.01, ***p≤0.001, ****p≤0.0001.

Approximately 35-40% of colorectal tumors exhibit mutations in *KRAS* gene. These mutations occur relatively early in the process of colorectal carcinogenesis [21–22]. Considering the pivotal role of SHP-2 in the activation of the RAS/RAF/MEK/ERK pathway, we examined whether SHP-2 is involved in intestinal epithelial transformation induced by oncogenic KRAS signaling [23–24]. We therefore developed recombinant lentiviruses encoding anti-*Shp-2* short hairpin RNA (shRNA) to stably suppress SHP-2 expression in rodent IECs transformed by oncogenic KRAS (IEC-6/KRAS^G12V^ cells) [23]. As demonstrated in Figure 2A, levels of SHP-2 protein were markedly diminished in both untransformed and KRAS-transformed IEC-6 cells, in contrast to a control shRNA which had no effect. SHP-2 knock-down significantly reduced the proliferation rate of IEC-6/KRAS cells without severely affecting proliferation of control cells (Figure 2B). In addition, SHP-2 silencing partially restored an epithelial morphology in KRAS-transformed cells (Figure 2C). Inhibition of SHP-2 expression also decreased the capability of KRAS cells to grow under anchorage-independent conditions (Figure 2D) and to invade Matrigel (Figure 2E). The ability of these cell populations to form tumor *in vivo* was subsequently assessed in immunodeficient mice. As illustrated in Figure 2F, KRAS cells induced palpable tumors rapidly, 8 days after injection. Of note, SHP-2 silencing clearly attenuated their tumorigenic potential.

**Figure 3:**
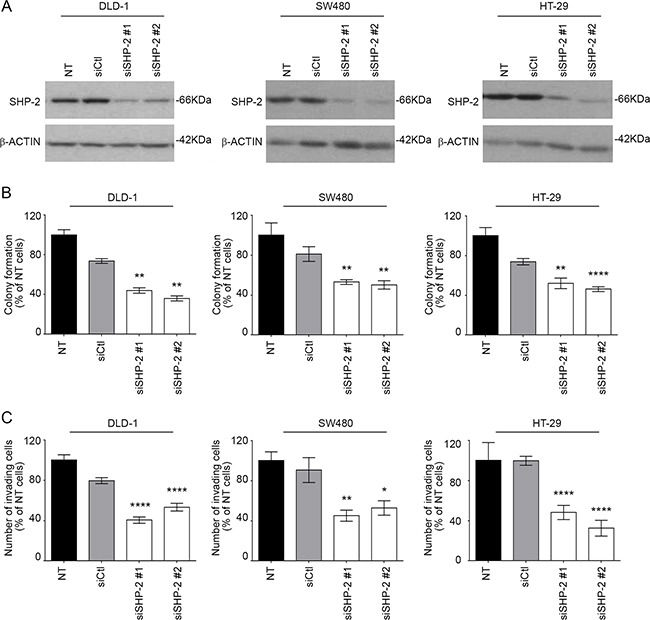
SHP-2 silencing in human CRC cells inhibits growth in soft agar and invasion capacity **A.** CRC cells were transfected or not (NT) with a control siRNA (siCtl) or encoding SHP-2-specific siRNAs (siSHP-2#1 and siSHP-2#2). Cell populations were lysed after 48h and protein lysates were analyzed by Western blotting for SHP-2 and β-actin expression. **B.** The capacity of cancer cells to grow in soft agar was assessed following SHP-2 silencing and the number of formed colonies counted after 7 to 14 days with DLD1, SW480 and HT29 cells. **C.** Invasion capacity was studied using Matrigel-coated Transwells after 48h post-seeding. The number of cells was determined in 10 fields and the experiments performed in duplicate. All experiments were conducted on at least three different cell populations. Data are expressed as mean ± SEM. *p≤0.05, **p≤0.01, ****p≤0.0001.

SHP-2 contribution was next analyzed in established human CRC cell lines, namely DLD-1 and SW480 which exhibit an activating mutation in the *KRAS* gene, and in HT-29 cells which have an activating *BRAF* mutation (Figure 3A). As shown in Figure 3B, cells transfected with siRNA against SHP-2 exhibited reduced capacity to form colonies in soft agarose and to migrate through Matrigel when compared to cells transfected with a control siRNA (Figure 3C).

### SHP-2 is required for full activation of MEK/ERK signaling in cells expressing oncogenic KRAS

**Figure 4:**
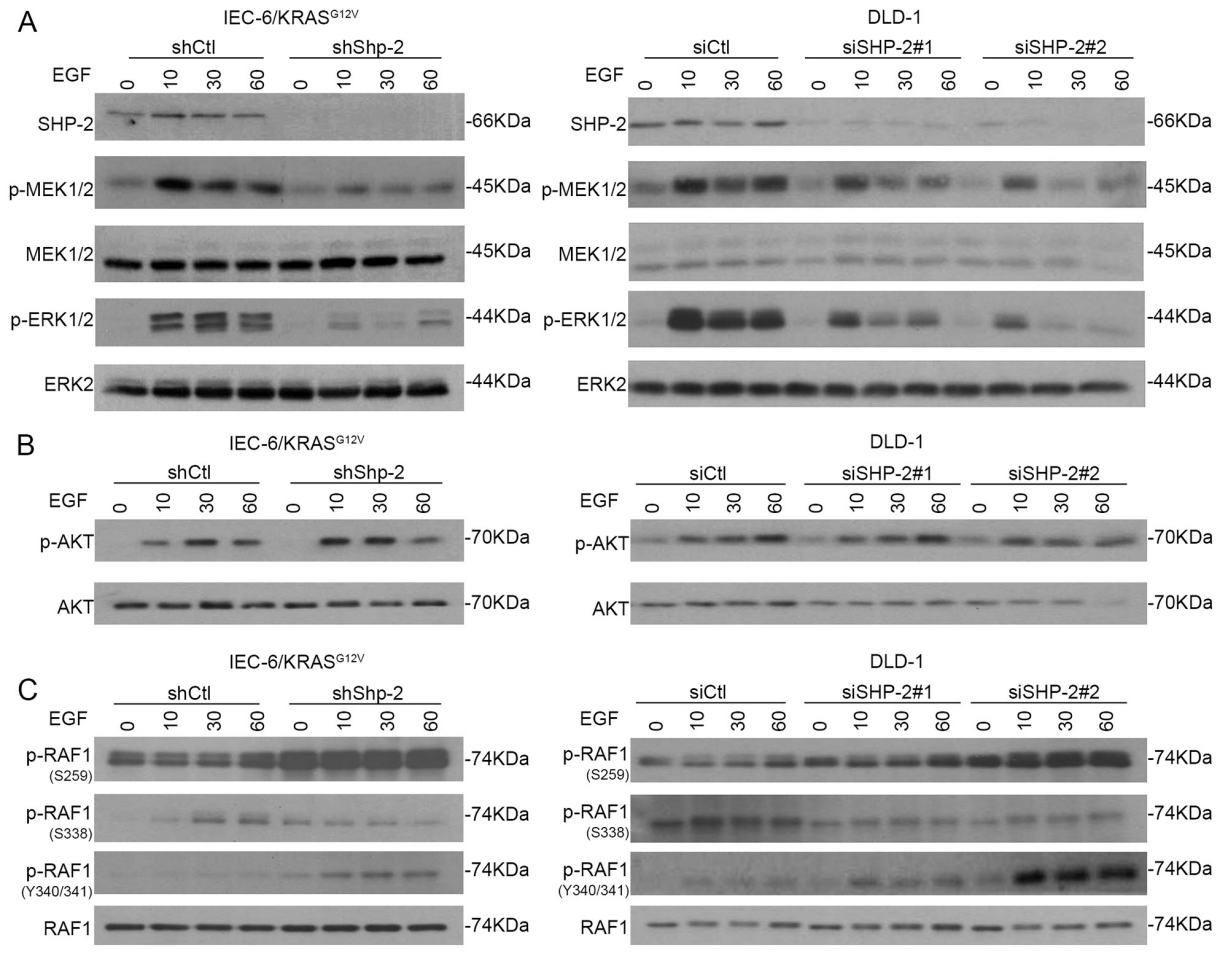
SHP-2 is required for full activation of MEK/ERK signaling in cells expressing oncogenic KRAS KRAS^G12V^ IEC-6 cells and DLD-1 CRC cells in which SHP-2 was downregulated or not by RNA silencing were serum-starved during 24h and thereafter stimulated with 100 ng/ml EGF for 10, 30 and 60 min. Cells were harvested and phosphorylation levels of MEK1/2 (S217/S221), ERK1/2 (T202/Y204) **A.** AKT (S473) **B.** and RAF-1 (S259/S338/Y340/Y341) **C.** were determined by Western blotting. All experiments were conducted on at least three different cell populations and representative immunoblots are shown.

To determine the mechanisms by which SHP-2 silencing interferes with the tumoral properties of cells expressing oncogenic KRAS, transduction pathways known to be associated with these oncogenes were examined, including the MAPK and PI3K/AKT effector pathways [25–26]. As shown in Figure 4A, activation of MEK1/2 and ERK1/2 by EGF was severely impaired in IEC-6/KRAS and DLD-1 cells knocked-down for SHP-2. By contrast, EGF-induced AKT activation was not significantly affected by SHP-2 silencing (Figure 4B).

Such reduced MEK phosphorylation observed in SHP-2-depleted cells suggests that SHP-2 silencing may interfere with activation of RAS or RAF. Interaction of RAF-1 with RAS has been shown to relieve RAF-1 auto-inhibition and is correlated with dephosphorylation of Ser259 and release of 14-3-3 inhibitory proteins [27]. Additionally, Ser338 and Tyr341 phosphorylation in the SSYY submotif is required for full activity of RAF-1 [27]. As seen in Figure 4C, increased phosphorylation of Ser259 was found in SHP-2 deficient cells. Of note, decreased phosphorylation of Ser338 was noticed in SHP-2-depleted cells while phosphorylation of Tyr340/341 was enhanced (Figure 4C). Since tyrosine phosphorylation of RAF proteins alone is not sufficient to activate the RAF kinase [28], our data strongly suggest that RAF-1 activation is likely impaired in SHP-2 deficient cells.

### SHP-2^E76K^ mutant does not act as a classical oncogene but is a novel modifier of intestinal adenoma formation

**Figure 5:**
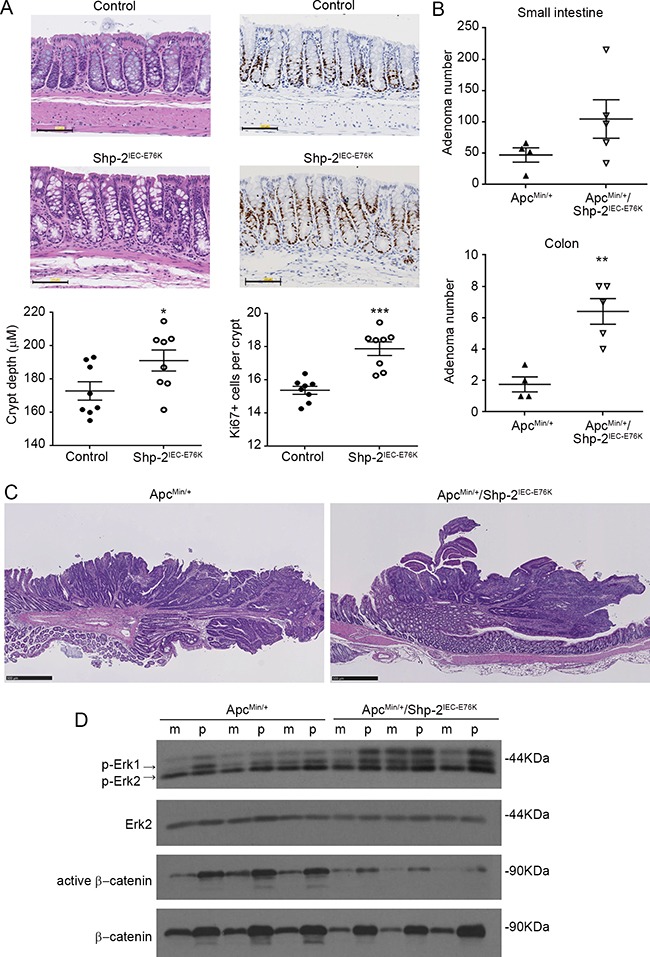
Expression of activated SHP-2^E76K^ mutant in intestinal epithelium deregulates crypt proliferation and increases tumor multiplicity in *Apc^Min/+^* mice **A.** Colonic sections obtained from 15 month-old control and *Shp-2^IEC-E76K^* mice were stained with H&E for histological analysis and KI67 for proliferation determination (N=8). Scale bars: 100 μm. **B.** The number of polyps formed in the small and large intestines was determined at 3 months of age in both *Apc^Min/+^* and *Apc^Min/+^* mice expressing activated SHP-2 mutant (*Apc^Min/+^; Shp-2^IEC-E76K^* mice) (N=4-5). **C.** Representative H&E staining of 3 month-old *Apc^Min/+^* and *Apc^Min/+^; Shp-2^IEC-E76K^* murine colonic polyps. Scale bars: 500μm. **D.** Western blot analyses were performed on paired specimens of margins (m) and polyps (p) extracted from *Apc^Min/+^* and *Apc^Min/+^; Shp-2^IEC-E76K^* mice. Levels of total and phosphorylated ERK1/2 (T202/Y204) and total and non-phospho (active) β-catenin were specifically analyzed. Representative immunoblots are shown. Data are expressed as mean ± SEM. *p≤0.05, **p≤0.01, ***p≤0.001.

Activating mutation in *PTPN11* gene (E76G) has been observed in certain CRCs [18, 29].To investigate the impact of somatic mutation on E76 residue *in vivo*, *Ptpn11^E76Kneo/+^/Villin-Cre^+^* mice (*Shp-2^IEC-E76K^* mice) were generated by crossing *Ptpn11^E76Kneo/+^* mice with *Villin-Cre* mice that express Cre in IECs. Neo deletion efficiency in IECs of these mice was almost complete and SHP-2^E76K^ expression was similar to that of wild-type SHP-2 in *Ptpn11^+/+^/Villin-Cre^+^* mice (data not shown). As illustrated in Figure 5A, IEC-specific expression of SHP-2^E76K^ mutant did not severely alter colonic architecture, except for a significant increase in crypt depth and proliferation (Figure 5A). Importantly, no change in colon phenotype of these mice was observed after 1 year, indicating that expression of activating SHP-2 mutation in IECs is not sufficient to induce their oncogenic transformation (Figure 5A). *Shp-*2^IEC-E76K^ mutant mice were then subsequently crossed with *Apc^Min/+^* mice, heterozygous for an *Apc* truncation mutation frequently found in human sporadic CRC, which spontaneously develop adenomas in the intestine [30]. Of note, *Apc^Min/+^* mice in C57BL/6 background have been shown to die before they reach four months due to anemia and intestinal obstruction resulting from polyposis [30]. Therefore, we sacrificed the mice at three months of age. Of note, the survival rate was similar between *Apc^Min/+;^ Shp-2^IEC-E76K^* and *Apc^Min/+^* mice at this age. As shown in Figure 5B, we observed that SHP-2^E76K^ expression in *Apc^Min/+^* mice significantly increased tumor multiplicity in the small and large intestines (Figure 5B). Histologically, both *Apc^Min/+^* and *Apc^Min/+^;Shp-2^IEC-E76K^* mice developed typical intestinal intraepithelial neoplasia (GIN) and adenomas (Figure 5C). As expected, polyps from *Apc^Min/+^* mice exhibited increased accumulation of β-catenin protein confirming the deregulation of APC signaling in these tumors (Figure 5D). Accordingly, levels of active (non-phosphorylated) β-catenin were significantly enhanced in polyps of *Apc^Min/+^* mice in comparison to normal tissues. Reduced levels of total and active β-catenin were however found in colonic epithelium and polyps from *Apc^Min/+^; Shp-2^IEC-E76K^* mice suggesting an attenuation of the Wnt/β-catenin signaling in these double mutant mice. Hence, pathways distinct of Wnt/β-catenin signaling most likely triggered these changes in colon tumor multiplicity. SinceMEK/ERK signaling activation has been deemedfundamental for intestinal tumor development in *Apc^Min/+^* mice [31], immunoblot analyses were performed to elucidate whether changes in ERK phosphorylation status can be observed in colon epithelial samples and polyps after expression of the activated SHP-2^E76K^ mutant. Analyses revealed that levels of p-ERK1/2 were indeed higher in colon epithelium and polyps of *Apc^Min/+^; Shp-2^IEC-E76K^* mice than in epithelium and polyps of control *Apc^Min/+^* mice.

### Shp-2^IEC-KO^ mice develop colorectal adenocarcinomas with age

**Figure 6:**
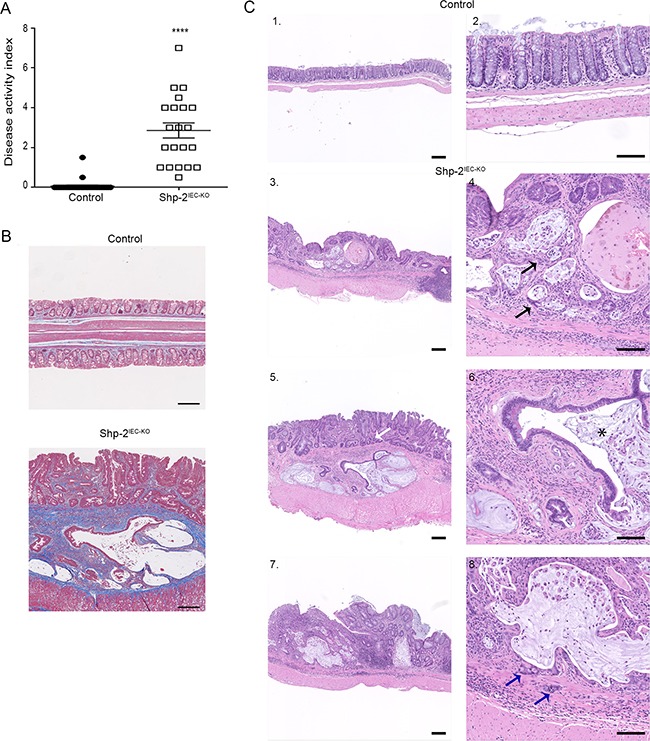
Mice with loss of epithelial SHP-2 expression develop colorectal adenocarcinomas with age **A.** Disease activity index of 15-month-old *Shp-2^IEC-KO^* mice and control littermates were calculated by scoring stool softness, occult fecal blood, rectal bleeding and colon rigidity (N=20). **B.** Masson's trichrome staining was performed to assess fibrosis in aged *Shp-2^IEC-KO^* mice and control littermates (N=6; blue=collagen fiber accumulation). **C.** Representative images of H&E staining of 15-month-old control and *Shp-2^IEC-KO^* murine colons (N=9; 100% of *Shp-2^IEC-KO^* mice had developed adenocarcinoma). Blue arrow shows cancerous cells invading the muscularis; black arrow shows cancerous cells in the mucosa; white arrow shows dysplasia; * indicates crypt abscess. Scale bars: 100μm. Data are expressed as mean ± SEM. ****p≤0.0001.

Persistent inflammation in the colon predisposes patients to CRC. Importantly, we previously reported that SHP-2 deletion in murine IECs (*Shp-2^IEC-KO^* mice) spontaneously leads to chronic inflammation in the colon, four weeks after birth [14]. Herein, *Shp-2^IEC-KO^* mice were followed longitudinally to evaluate their phenotype 15 months after birth. As shown in Figure 6, *Shp-2^IEC-KO^* mice still exhibited chronic colitis as visualized by the sustained elevated DAI (Figure 6A) and fibrosis (Figure 6B). This persistent inflammation further prompted to investigate whether *Shp-2^IEC-KO^* mice had developed colorectal tumors with age. As shown in Figure 6C, 15 months after birth, *Shp-2^IEC-KO^* mice had developed severe dysplasia and infiltrating adenocarcinomas in their distal colon. Some of these mice also developed invasive carcinomas as witnessed by invasion of tumor cells through the lamina propria (Figure 6C, panels 4 and 8, arrows).

**Figure 7:**
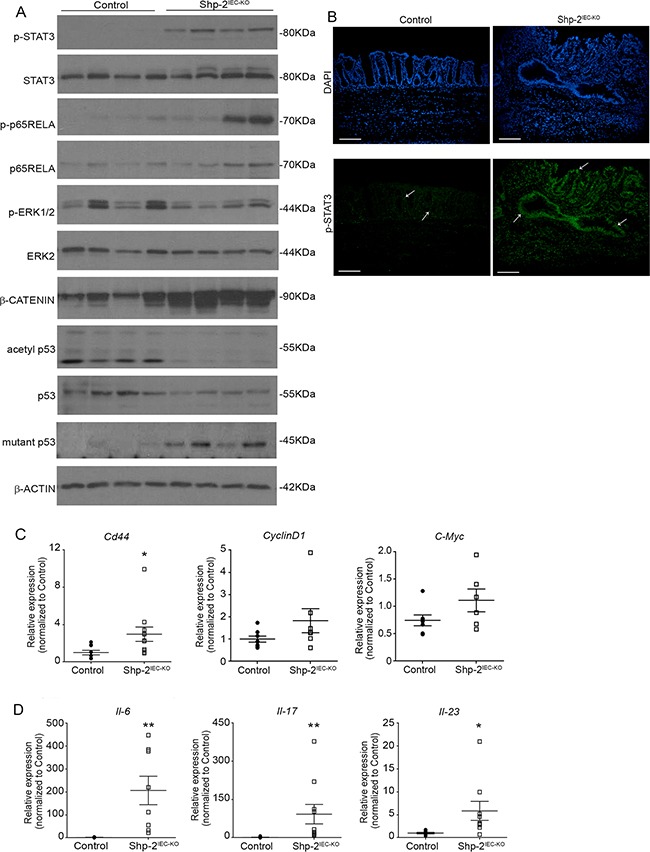
Aged *Shp-2^IEC-KO^* mice exhibit several typical features of colitis-associated cancer **A.** Mucosal enrichments from 15 month-old *Shp-2^IEC-KO^* and control murine colons (N=8) were analyzed by Western blotting for the expression of selected proteins including total and phosphorylated ERK1/2 (T202/Y204), STAT3 (Y705) and p65NFκB (S536) as well as total β-catenin, total p53, mutant-p53 (R273H) and acetylated p53 (K379). β-actin served as loading control. Representative immunoblots are shown. **B.** Immunofluorescence against phosphorylated STAT3 (Y705) was performed on colonic tissue from 15 month-old control and *Shp-2^IEC-KO^* mice. White arrows indicate epithelial cells. Scale bars: 100μm. **C.** Quantitative PCR of *Cd44*, *Cyclin D1* (p=0.1385) and *C-Myc* (p=0.1254) mRNAs in enriched colonic mucosal extracts from 15-month-old control and *Shp-2^IEC-KO^* mice were performed. Relative expression was normalized with the housekeeping genes *Psmc4, Pum1* and *Tbp* (N≥6). **D.** Quantitative PCR of interleukin-6 (*Il6*), interleukin-17 (*Il17*) and interleukin-23 (*Il23*) mRNAs in enriched colonic mucosal extracts from 15 month-old control and *Shp-2^IEC-KO^* mice were performed. Relative expression was normalized with the housekeeping genes *Psmc4*, *Pum1* and *Tbp* (N≥8). Data are expressed as mean ± SEM. *p≤0.05, **p≤0.01.

To understand the molecular mechanisms involved in tumor development in aged *Shp-2^IEC-KO^* mice, we analyzed the activation levels of signaling effectors known to be regulated by SHP-2 and to be involved in chronic intestinal inflammation. As illustrated in Figure 7A, the downstream transcription factors STAT3 and p65NFκB, both of which are tumor-promoting factors, were clearly phosphorylated and activated in the colons of *Shp-2^IEC-KO^* mice. Immunofluorescence confirmed increased STAT3 phosphorylation in the colon from these mice, especially in epithelial cells (Figure 7B, see arrows). Interestingly, while the expression of acetylated p53 was barely detectable, levels of mutant p53 (R273H) were clearly increased in *Shp-2*-deficient colons (Figure 7A), indicating that *Tp53* gene was targeted during colitis-induced carcinogenesis. Moreover, phosphorylation levels of ERK1/2 MAPK were reduced in aged *Shp-2 KO* mice (Figure 7A) as previously observed in younger mice [14]. By contrast, expression levels of β-catenin protein (Figure 7A) as well as *Cd44*, *CyclinD1* and *C-Myc* transcripts (Figure 7C) were enhanced in the colon of mutant mice suggesting the activation of Wnt/β-catenin signaling. Lastly, we analyzed the expression of proinflammatory cytokines IL-6, IL-17 and IL-23 recently shown to influence the development and growth of colitis-associated cancer (CAC) [32–35]. As shown in Figure 7D, levels of these cytokines were indeed significantly increased in aged *Shp-2^IEC-KO^* mice.

**Figure 8:**
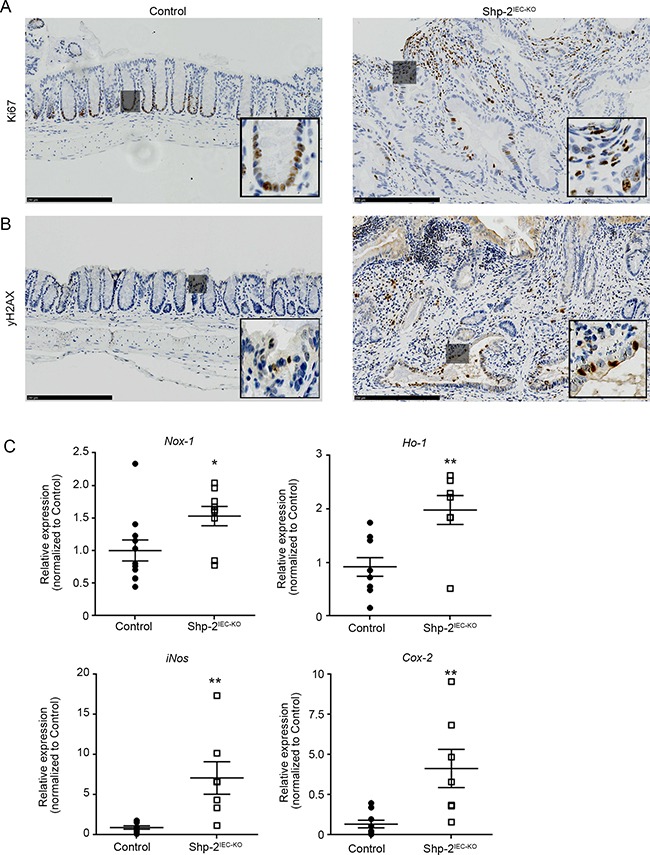
Aged *Shp-2^IEC-KO^* mice exhibit increased epithelial proliferation and signs of oxidative stress in their colon **(A-B)** Representative images of 15 month-old control and *Shp-2^IEC-KO^* murine colons stained for KI67 (A) and phospho-H2AX (B) are shown (representative of N=6). Scale bars: 250μm. **C.** Quantitative PCR of *Nox1*, *Ho-1*, *iNos* and *Cox-2* mRNAs in enriched colonic mucosal extracts from 15-month-old control and *Shp-2^IEC-KO^* mice were performed. Relative expression was normalized with the housekeeping genes *Psmc4, Pum1* and *Tbp* (N=9). Date are represented as mean ± SEM. *p≤0.05, **p≤0.01.

Immunohistochemistry analysis furthermore demonstrated increased KI67 staining, a marker of proliferation, especially in the nucleus of most epithelial and mesenchymal cells in dysplastic regions of aged *Shp-2^IEC-KO^* mice while its expression was restricted to the nucleus of proliferative progenitor cells along the colonic crypt in control mice (Figure 8A). Increased phospho-H2AX staining (Figure 8B) as well as *Nox1*, *Ho-1*, *iNos* and *Cox2* expression (Figure 8C) were moreover observed in the colon of 15 month-old *Shp-2^IEC-KO^* mice indicating the presence of DNA damage and oxidative stress, two typical features of CAC [36–37]. Overall, these results suggest that SHP-2 protects the colonic epithelium against inflammation-induced cell proliferation and DNA damage.

### IEC-specific Shp-2 ablation exacerbates tumorigenesis in Apc^Min/+^ mice

**Figure 9:**
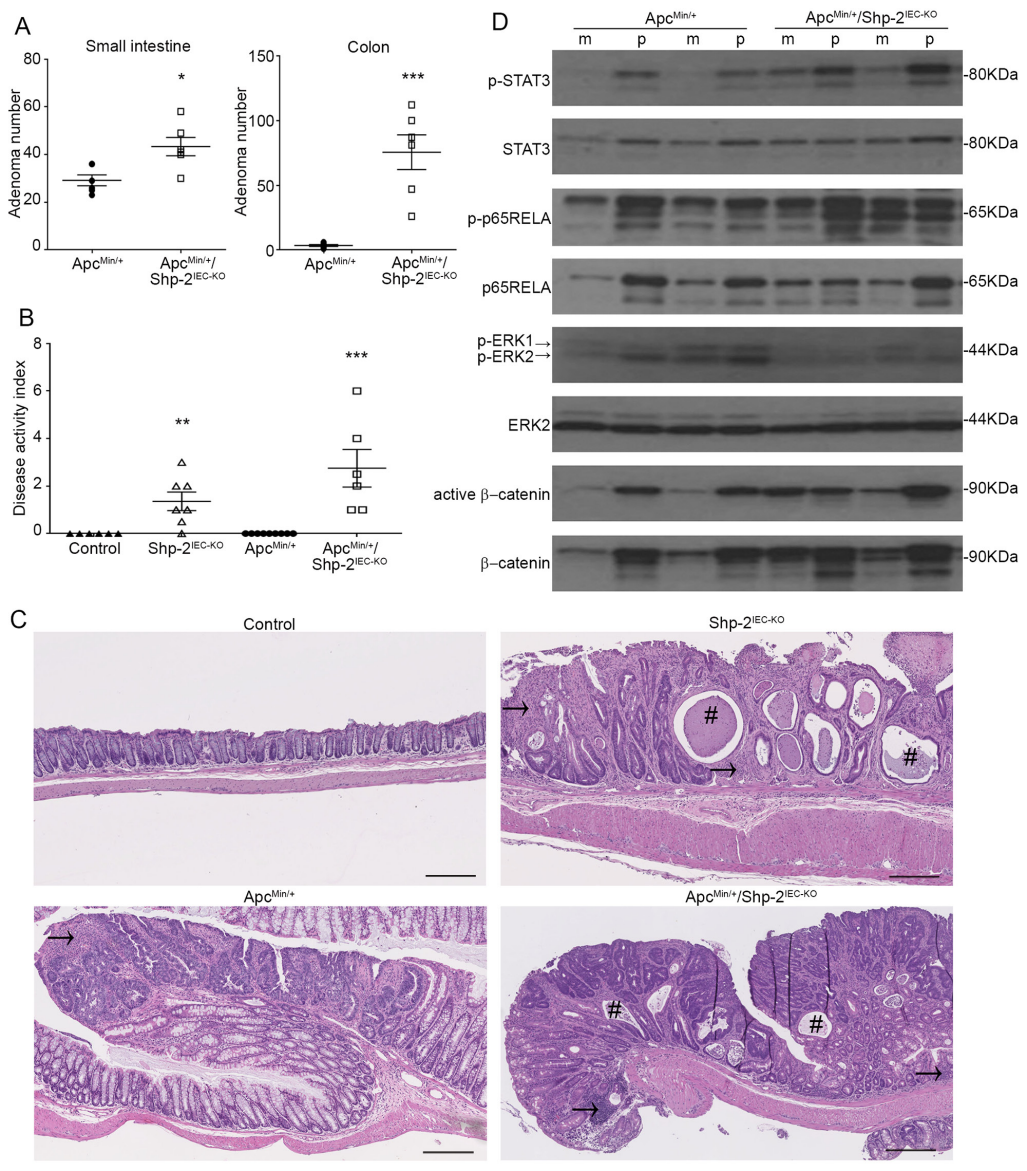
IEC-specific *Shp-2* ablation exacerbates tumorigenesis in *Apc^Min/+^* mice **A.** The number of polyps formed in the small intestine and colon was determined at 3 months of age in both *Apc^Min/+^* and *Apc^Min/+^* mice lacking Shp-2 expression (*Apc^Min/+^*; *Shp-2^IEC-KO^* mice) (N=6). **B.** Disease activity index of 3 month-old mice was calculated by scoring stool softness, occult fecal blood, rectal bleeding and colon rigidity (N=6). **C.** Representative images of 3 month-old control *Shp-2^IEC-KO^*, *Apc^Min/+^* and *Apc^Min/+^; Shp-2^IEC-KO^* murine colons. # indicates crypt abscesses and arrow shows immune cell infiltration. Scale bars: 100μm. **D.** Western blot analyses were performed on paired specimens of margins (m) and polyps (p) extracted from *Apc^Min/+^ and Apc^Min/+^; Shp-2^IEC-KO^* mice. Levels of total and phosphorylated ERK1/2 (T202/Y204), STAT3 (Y705) and p65NFκB (S536) as well as total and non-phospho (active) β-catenin were specifically analyzed. Representative immunoblot are shown. Data are expressed as mean ± SEM. *p≤0.05, **p≤0.01, ***p≤0.001.

To further analyze the suppressive function of SHP-2 in inflammation-induced intestinal tumorigenesis, the effect of SHP-2 removal was assessed in *Apc^Min/+^* mice. As illustrated in Figure 9A, the multiplicity of polyps after SHP-2 epithelial loss was much more dramatic in the colon than in the small intestine of *Apc^Min/+^*;*Shp-2^IEC-KO^* mice comparatively to control *Apc^Min/+^* mice at three months of age. Importantly, 38% of *Apc^Min+^;Shp-2^IEC-KO^* mice died before three months, probably because of malnutrition caused by severe diarrhea and rectal bledding. Indeed, at this age, *Shp-2^IEC-KO^* and *Apc^Min/+;^Shp-2^IEC-KO^* mice have clearly developed chronic colitis as demonstrated by higher DAI in comparison to control littermates (Figure 9B). Histological analysis confirmed the increased immune cell infiltration, the reduction in goblet cells and the presence of crypt abscesses in *Shp-2*-deficient colons (Figure 9C). Importantly, these signs of inflammation were also manifest in the colon of *Apc^Min/+^*;*Shp-2^IEC-KO^* mice (Figure 9C), correlating with elevated DAI similar to *Shp-2^IEC-KO^* mice (Figure 9B).

In comparison to their corresponding benign epithelium, polyps from *Apc^Min/+^* mice displayed consistently higher phosphorylation levels of ERK1/2, STAT3 and p65RELA (Figure 9D). Importantly, upon deletion of SHP-2 in *Apc^Min/+^* mice, phosphorylated levels of ERK1/2 were found to be diminished both in the polyps and margins in comparison to *Apc^Min/+^* mice. By contrast, total and active levels of β-catenin, STAT3 and p65RELA were further increased in the colon of *Apc^Min/+^*; *Shp-2^IEC-KO^* mice. These observations suggest that SHP-2 may exert a tumor suppressive function under an inflammatory setting.

## DISCUSSION

Genetic and biochemical evidence strongly demonstrates that SHP-2 is essential for full activation of RAS/ERK MAPK pathway by most receptors [38–39]. Furthermore, activating mutations in *PTPN11* gene have also been described in the developmental disorder Noonan syndrome and in pediatric leukemias [40]. Likewise, somatic *PTPN11* mutations were found in certain solid tumors such as lung, liver and CRCs [18]. These studies therefore suggest that SHP-2 may be a bona fide oncoprotein [19].

Consistent with the above, SHP-2 was found to be overexpressed in gastric cancer, breast cancer and non-small cell lung cancer (NSCLC) [40–42]. However, there have been sparse reports regarding SHP-2 expression in CRC [43]. Recently, Cai et al. (2014) analyzed SHP-2 levels in 232 unpaired tumor specimens from patients with CRC. They found that low SHP-2 expression correlated with poor tumor differentiation, late TNM stage and lymph node metastasis. Herein, we found significant increase in SHP-2 mRNA and protein levels in colorectal adenomas and stage 1 tumors. Notably, the increased expression of SHP-2 was primarily detected in the hyperplastic epithelium and not in the lamina propria, indicating that SHP-2 may be involved early in the development of sporadic colorectal tumors. However, in more advanced CRCs, SHP-2 mRNA levels were not significantly modulated in comparison to their corresponding margins. We verified *PTPN11* gene promoter methylation in human colon specimens from the TCGA database (TCGA Research Network: http://cancergenome.nih.gov), interrogated and plotted using the TCGA Wanderer interface [44]. Overall, we did not find significant difference in *PTPN11* gene promoter methylation suggesting that it is not the mechanism by which *SHP-2* gene expression is regulated in advanced CRCs (data not shown). Since the epithelium to stroma ratio varies and changes considerably during tumor progression, one might speculate that this may influence protein/RNA quantification. It would be pertinent in the future to perform immunohistochemistry analyses in advanced tumors to verify SHP-2 expression in epithelial cells versus the surrounding stromal tissue.

Up to 60% of colorectal tumors exhibit gain-of-function mutations in *KRAS, NRAS and BRAF* genes [21–22]. These mutations generally occur early in the adenoma-carcinoma sequence [21], triggering cell transformation, hyperproliferation and anoikis resistance [45–46]. Accordingly, most of colorectal adenomas analyzed herein exhibited *KRAS* (G12D, G13D, Q61H) or *BRAF* (V600E) activating mutations, in combination with *APC* inactivating mutations (exon 15) [24]. Considering the significance of SHP-2 in the control of RAS pathway, we speculated that this phosphatase likely plays an important role in colorectal carcinogenesis. Indeed, we found that SHP-2silencing in KRAS-transformed IECs severely impaired both their proliferation rate and invasive capabilities. Moreover, cells displaying knocked-down SHP-2 exhibited reduced tumor formation capacity. Similarly, targeting SHP-2 by RNAi in human KRAS or BRAF-mutated established CRC cells limited their ability to grow in soft agar and to invade Matrigel. Interestingly, these inhibitory effects of SHP-2 silencing were associated with reduced phosphorylation of RAF, MEK and ERK proteins in cells expressing oncogenic KRAS. At first glance, these data are in agreement with those demonstrating that the phosphatase activity of SHP-2 is indeed necessary for full RAS activation. Accordingly, cells expressing dominant-negative SHP-2 [38] or *Ptpn11* gene exon 3-deleted mouse embryonic fibroblasts [39] exhibit defective RAS activation. Furthermore, SHP-2 may also function either downstream and/or in parallel to RAS since other data have shown that SHP-2 inhibition can impair the activation of downstream effectors even in the presence of oncogenic RAS [47]. Recent results have further demonstrated that SHP-2 binds and dephosphorylates both wild-type and oncogenic HRAS on Tyr32 in astrocytes and glioma cells, an event that increases RAS association with RAF-1 and downstream MEK/ERK activation [48]. Herein, dysregulated phosphorylation of RAF-1 was observed in SHP-2 deficient cells expressing oncogenic KRAS. Since Tyr32 is conserved in human and mouse KRAS proteins, our data suggest that SHP-2 may also target KRAS in IECs, enhancing the stimulation of downstream proliferative RAS/MEK/ERK kinases.

The somatic E76G mutation specifically found in colorectal cancer specimens disrupts the inhibitory intramolecular interaction within the PTP domain and leads to the hyperactivation of SHP-2 [40]. Given the latter, we speculated that by inducing the RAS/ERK signaling pathway, expression of the SHP-2 E76 mutant in IECs would be sufficient to trigger intestinal neoplasia. However, *Ptpn11* E76K knock-in mice failed to develop any intestinal tumors after 1 year. These findings hence indicate that SHP-2 does not function as a classical oncogene in intestinal epithelium. SHP-2 may need to cooperate with additional oncogenic events such as the loss of the *Apc* gene to initiate intestinal neoplasia. Our findings hence support the model in which constitutive activation of SHP-2 drives the development of adenoma in the intestine of *Apc^Min/+^* mice. SHP-2 expression appears to be required for full activation of ERK1/2 both in normal intestinal epithelium [14] and *Apc*-mutated epithelium (Figure 9D). An important role of the ERK pathway in the establishment of the Min phenotype has moreover been previously described [31]. Therefore, in addition to the inactivation of *Apc*, activation of ERK signaling, either by the activation of SHP-2 as demonstrated herein or by mutation of KRAS [49], appears to act synergistically resulting in increased tumor multiplicity in the intestine.

In addition, we further demonstrate that mice with conditional deletion of *Shp-2* in IECs developed colorectal adenocarcinomas with age. These adenocarcinomas exhibited several features that distinguish colitis-associated cancer including mucosal ulcerations, regenerative proliferation and persistent histological inflammation [50]. Likewise, signs of oxidative stress (increased *Nox1*, *Ho-1*, *iNos and Cox-2*) and DNA damage (phospho-H2AX) were also observed in the colon of aged *Shp-2^IEC-KO^* mice. A major mechanism that links inflammation to proneoplastic genetic alterations involved in the pathogenesis of CAC is oxidative stress [50]. Oxidative stress is mainly produced by cells of the innate immune system such as macrophages and granulocytes which generate various reactive oxygen and nitrogen species (ROS, NOS) which impose a persistent mutational pressure to IECs, resulting in DNA breaks and adducts. In this regard, base transversions in *TP53* gene were often observed in UC patients who develop CAC, and oxidative DNA damage may presumably be responsible for these errors [36,51]. Accordingly, increased levels of the mutant form of p53 (R273H), a hot spot residue in CRC [52], were detected in inflamed mucosae from aged *Shp-2^IEC-KO^* mice. Moreover, in recent years, major pro-inflammatory pathways have been implicated in inflammation-associated tumor development. Among these, the transcription factors NFκB and STAT3 have taken center stage [16–17]. Indeed, Greten *et al.* showed that deletion of *IKKβ* in IECs resulted in decreased numbers of tumors and increased apoptosis in the azoxymethane (AOM)-dextran sodium sulfate (DSS) model [53]. Besides, specific STAT3 ablation in IECs interfered with tumor formation and tumor growth in the AOM-DSS model [33]. Conversely, IEC-specific *Socs3* ablation or expression of the SOCS3-binding deficient *gp130^Y757F^* mutation both inducing STAT3 activation, promoted colonic tumor growth and incidence [33, 54]. Thus, tumor cell-specific NFκB and STAT3 signalings appear to be required for inflammation-associated tumor initiation in the colon [2], likely by inducing the expression of pro-proliferative, anti-apoptotic and immune response genes [33]. Furthermore, activated NFκB can suppress p53 acetylation and transactivation through sequestration of CBP or p300 [55], inhibiting p53 tumor suppressor function [56]. Interestingly, NFκB and STAT3 have both been shown to be hyperphosphorylated in the colonic epithelium of 1-month [14] and 15-month-old *Shp-2^IEC-KO^* mice. Furthermore, levels of acetylated p53 were markedly downregulated in the colon of these mice, thereby creating permissive conditions for oncogenic transformation. The exact mechanism by which *Shp-2* ablation in IECs induces NFκB and STAT3 activation is not totally clear. However, we can speculate that this is an IEC-intrinsic consequence of *Shp-2* inactivation since SHP-2 silencing in cultured IECs increases the levels of phosphorylated STAT3 and p65RELA [14]. Furthermore, SHP-2 can closely regulate phosphorylation and activation of both of these transcription factors in several cell types [9].

Paradoxically, activation of NFκB and STAT3 in IECs specifically has been demonstrated to protect the gut against inflammation. Indeed, conditional IEC-specific deletion of NEMO (essential for NFκB activation) spontaneously caused colon inflammation [57]. Likewise, mice with deletion of STAT3 in IECs showed increased susceptibility to DSS [58] and to *Citrobacter rodentium* infection [59]. Hence, both NFκB and STAT3 expressed in IECs exert a protective, homeostatic function under physiological conditions [57]. Therefore, this indicates that *Shp-2^IEC-KO^* mice develop colitis despite the rapid and persistent activation of STAT3 and NFκB in IECs. We cannot exclude however that excessive activation of STAT3 and/or NFκB might also contribute to the pathogenesis of the inflammatory phenotype in *Shp-2^IEC-KO^* mice [60–61]. Nevertheless, by inducing chronic inflammation as well as STAT3 and NFκB activation, IEC-specific deletion of *Shp-2* results in the formation and growth of pre-malignant and malignant lesions typical of colitis-associated carcinogenesis.

Our data also reveal that SHP-2 inactivation in IECs considerably exacerbated mutant *Apc*-initiated transformation, notably shifting tumor incidence toward the colon. Similarly, 1-week treatment of *Apc^Min/+^* mice with Dextran Sulfate Sodium (DSS), which causes acute colonic inflammation, was found to markedly increase the multiplicity and incidence of colonic neoplasms in these mice [62]. These findings thus reinforce the notion that SHP-2 exerts a tumor suppressive action in the colon by inhibiting the development of inflammation [14], probably through the maintenance of appropriate colonic barrier function [63]. Such anti-oncogenic action of SHP-2 was also recently described in a mouse model of liver cancer [15] in which mice with hepatocyte-specific deletion of *Shp-2* developed severe inflammation in the liver leading to tumor development.

Our study thus demonstrated that both activation and inactivation of Shp-2 in mouse colonic epithelium promotes tumor development. Such dual role for Shp-2 in cancer development was also previously proposed in the liver [64–65]. However, it is important to emphasize that inactivation of Shp-2 in the colon epithelium was much more detrimental for the mice than the activation of Shp-2. Indeed, *Shp-2^IEC-KO^* mice showed severe diarrhea and rectal bleeding with higher mortality in comparison to controls. By contrast, no difference on survival rate was noticed between *Shp-2^IEC-E76K^* mice and their control littermates. Furthermore, all *Shp-2^IEC-KO^* mice developed colorectal adenocarcinomas with age; however, no *Shp-2^IEC-E76K^* have developed intestinal neoplasia after 15 months of age. Lastly, colonic tumor multiplicity in *Apc^Min/+^* mice was much more increased by *Shp-2* deletion (35-fold) than by *Shp-2* activation (3-fold).

Although the molecular mechanisms underlying the opposing actions of SHP-2 in IECs remain to be clarified, they may predominantly rely on the ability of SHP-2 to activate the ERK signaling. Indeed, the ERK pathway, which is consistently stimulated upon activation of SHP-2 [8–10, 14], is commonly associated with intestinal crypt cell proliferation and tumorigenesis [31, 66]. Conversely, activation of ERK in secretory progenitors has been recently shown to direct Goblet cell differentiation [63,67] and these cells are crucial for the maintenance of barrier function and the prevention of intestinal inflammation [68]. More specifically, Heuberger *et al*. also found that ERK signaling controls secretory cell fate in the small intestine by negatively regulating Wnt/β-catenin activity. Unfortunately, they did not analyze β-catenin protein expression but they showed, in intestinal organoids and cultured cells, that ERK inhibition changed the relative abundance of Tcf4 isoforms [67]. How exactly SHP-2 regulates β-catenin protein levels remains therefore to be examined. Intriguingly, we previously reported that ectopic expression of SHP-1, a SHP-2 paralog, stimulates β-catenin proteasomal degradation, decreasing its transcriptional activity [69]. Current experiments are in progress to verify if SHP-2 can similarly regulate β-catenin stability and activity in cultured IECs.

In conclusion, the present study reveals that SHP-2 exerts opposing functions, pro-oncogenic versus anti-oncogenic (by inhibiting inflammation), in the colon epithelium. These opposing functions for SHP-2 may depend on cellular context (transit-amplifying cells, secretory progenitor cells). Further studies are required to identify the binding partner proteins of SHP-2 that may direct these opposing cellular responses under physiological and pathological conditions.

## MATERIAL AND METHODS

### Materials

*Antibodies.* Primary antibodies were obtained from the following sources: SHP-2, MEK1/2, ERK2, phospho-RAF1 (Y340/Y341), RAF1, KI67 and γH2AX from Santa Cruz Biotechnology (Santa Cruz, CA, USA), phospho-ERK1/2 (T202/Y204) from Sigma-Aldrich (Mississauga, ON, Canada), β-actin from Chemicon International (Billerica, MA, USA), GAPDH (HRP conjugate), phospho-RELA (S536), RELA, phospho-STAT3 (Y705), STAT3, phospho-RAF-1 (S259), phospho-RAF-1 (S338), non-phospho (active) β-catenin, phospho-AKT (S473), AKT, acetylated-p53 (K379) and phospho-MEK1/2 (S218/S222) from Cell Signaling Technology (Danvers, MA, USA), total p53 and mutant-p53 (R273H) from Abcam (Toronto, ON, Canada) and β-catenin from BD Pharmingen (Mississauga, ON, Canada). Mouse and rabbit horseradish peroxidase antibodies were purchased from Amersham Biosciences (Pittsburg, PA, USA). All other materials were from Sigma-Aldrich (Oakville, ON, Canada) unless stated otherwise.

### Human colorectal tissues

Ninety four specimens of colon tumors and paired normal tissues (at least 10 cm from the tumor) were obtained from patients undergoing surgical resection and were processed as previously described [70]. Patients did not receive neoadjuvant therapy. Tissues were collected after obtaining the patient's written informed consent, according to the protocol approved by the Institutional Human Subject Review Board of the Centre Hospitalier Universitaire de Sherbrooke. Pathological and clinical data were obtained from medical records and are provided in Supplementary Table S1.

### Cell culture

The rat intestinal epithelial crypt cells IEC-6 stably overexpressing empty vector (pBabepuro) or KRAS^G12V^ mutant were generated after retroviral infection and were previously characterized and cultured as described [23]. The colon carcinoma cell lines SW480, HT29 and DLD-1 were cultured as described [70–71]. HT-29 and SW480 cell lines were purchased from ATCC (Manassas, VA, USA) in October 2014 and DLD-1 were authenticated using Cell authentication service from ATCC in October 2014.

### Generation of shRNA, production of lentiviruses production and cell infection or transfection

The lentiviral shRNA expression vector (pLenti6-U6) was constructed as described [60]. Sequences of rat shRNA oligonucleotides are available upon request [14]. Irrelevant pLenti-shRNA with the scrambled rat shShp-2 sequence was used as negative control (shControl). Lentiviruses produced in 293T cells were used for infection of IEC-6 cells according to Invitrogen recommendations. To knock down SHP-2 in human colorectal cancer cells, cells were transfected using Lipofectamine 2000 (Invitrogen, Waltham, MA, USA) with 50nMol SHP-2 siRNA corresponding to fw5′-UAAAUCGGUACUGUGCUUCUGUCUG-3′ and rev5′- CAGACAGAAGCACAGACCGAUUUA-3′ for siRNA#1 or to fwd5′-AAUAUUUGUAUAUUCGUGCCCUUUC3′ and rev5′GAAAGGGCACGAAUAUACAAAUAUU-3′ for siRNA#2 (IDT, Coralville, IA, USA) as described by Wang HC et al. [72]. A scrambled siRNA was also used as a control (siCTL-Invitrogen Stealth™ RNAi Negative Control LOGC).

### Western blot analysis

Protein extractions and Western blot analyses were performed as described [14, 24].

### Cell proliferation, soft agarose and migration/invasion assays

Experiments were started 7 days post-selection (for pLenti-shRNA infection) or 48 hours post transfection (for RNAi). For growth assays, cells were seeded in 6-well plates (3.5×10^4^cells/well). The number of cells was calculated daily during 7 days using a Cell particle counter (Invitrogen). Soft agarose and migration/invasion assays (Biocoat Matrigel, Becton Dickinson, Bedford, MA, USA) were performed as reported [70]. Cells were visualized by inverted light microscopy (Zeiss apparatus) and crystal violet stained inserts were visualized by light microscopy (Leica apparatus). Cell count was obtained with ImageJ software. For all experiments, three different cell populations originating from three different infections (shRNA) or transfections (siRNA) were analyzed.

### Immunocompromised mice

CD1 nu/nu mice were purchased from Charles River Laboratory (Wilmington, MA, USA). Mice were housed in individually-ventilated cages. Food, water, bedding and cages were sterilized. Experiments were approved by the Animal Research Committee of the Faculty of Medicine and Health Sciences of the Université de Sherbrooke. A total of 5×10^5^ cells suspended in 0.1 ml DMEM were injected into the dorsal subcutaneous tissue of 7-week-old female mice. Tumor volume was determined every 3 days by external measurement according to the formula (*d*^ 2^ × *D*)/2. Three different cell populations originating from three different viral infections and different virus productions were assessed in minimally four different mice per population, for a total of 13 mice.

### Conditional KO and KI mice

*Shp-2* floxed allele mice [14] were previously crossed with *Villin*-Cre mice (Jackson Laboratory) to specifically delete *Shp-2* in the intestinal epithelium (referred as *Shp-2^IEC-KO^*) [14]. Knock-in *Ptpn11^E76K/+^* mice [29] (on C57BL6/J background) were crossed with *Villin*-Cre mice to specifically express heterozygote *Shp-2^E76K/+^* mutant in the intestinal epithelium (referred as *Shp-2^IEC-E76K^*). Only homozygote *Shp-2^+/+^* mice were used as control littermates. C57BL6/J-*Apc*^Min/+^ mice were obtained from Jackson Laboratory (Bar Harbor, ME, USA). These mice were mated to generate *Apc^Min/+^*; *Shp-2^IEC-KO^* and *Apc^Min/+^; Shp-2^IEC-E76K^* models. The first cross was between the *Villin-Cre* and *Apc^Min/+^* mice. The double heterozygous mice were then bred with *Shp2*^flox/flox^ mice one time and the triple heterozygous mice were finally bred with *Shp2*^flox/flox^ mice to generate the experimental mice (*Apc^Min/+^*; *Shp-2^IEC-KO^*) and control littermates. The double heterozygous *Apc^Min/+^; Villin*-Cre mice were also bred with *Ptpn11^E76K/+^* mice to generate the triple heterozygous experimental mice and their control littermates. Genomic DNA was isolated using the Spin Doctor genomic DNA kit from Gerard Biotech following the manufacturer's protocol. For the knock-in *Shp-2^IEC-E76K^ mice*, loss of *Neo* cassette was measured by PCR analysis to validate the *Shp*-2^E76K^ expression in intestinal tissue [29]. PCR conditions and primer sequences used for genotyping are available upon request. All experiments in mice were approved by the Animal Research Ethics Committee of the Faculty of Medicine and Health Sciences of the Université de Sherbrooke.

### Histological staining, immunohistochemistry and macroadenoma counts

Colons were fixed, sectioned and stained as described previously [73]. Fibrosis was visualized using Trichrome Stain (Masson) Kit (Sigma-Aldrich). Polyps were counted and measured in the gastrointestinal tract from stomach to rectum of control and mutant *Apc^Min/+^* littermates as previously described [73–74].

### RNA extraction and quantitative RT-PCR analysis

RNA was isolated from the scraped colonic mucosa of mice using the RNeasy minikit (Qiagen). RT-PCR was performed using AMV-RT (Roche Diagnostics) according to the manufacturer's instructions and quantitative PCR was performed by the RNomics Platform at the Université de Sherbrooke (Sherbrooke, QC, Canada). All primer sequences and cycling conditions are available upon request.

### Statistical analysis and figure editing

All assays were minimally performed in triplicate. Typical results shown are representative of 3 independent experiments and data are expressed as mean ± SEM. Densitometric analysis was performed using ImageJ software. Results were analyzed using the Student's *t*-test and were considered statistically significant at *p* ≤ 0.05. Graphs and statistics were generated with Graphpad Prism (Graphpad Software Inc., LaJolla, CA) and figures were constructed using Photoshop software.

## SUPPLEMENTARY MATERIAL TABLE



## Abbreviations

AOM: azoxymethane
APC: adenomatous polyposis coli
CAC: colitis-associated cancer
Cox-2: cyclooxygenase-2
CRC: colorectal cancer
DSS: dextran sulfate sodium
EGF: epidermal growth factor
ERK: extracellular signal regulated kinase
IBD: inflammatory bowel diseases
IEC: intestinal epithelial cell
IKKβ: IκB kinase
iNOS JAK: Janus-associated kinase
JNK: c-jun N-terminal kinase
MAPK: mitogen-activated protein kinase
NFκB: nuclear factor kappa B
NOS: nitrogen oxygen species
NSCLC: non-small cell lung cancer
PI3K: phosphatidylinositol 3-kinase
RELA: v-rel reticuloendotheliosis viral oncogene homolog A
ROS: reactive oxygen species
Ser: serine
SHP-2: Src homology 2-domain-containing phosphatase
shRNA: short hairpin RNA
SOCS3: suppressor of cytokine signaling 3
STAT: signal transducer and activator of transcription
Tyr: tyrosine
UC: Ulcerative colitis
