# Supplementary material for: SHP-2 phosphatase contributes to KRAS-driven intestinal oncogenesis but prevents colitis-associated cancer development
Source: Oncotarget. 2016 Aug 25;7(40):65676–95. doi: 10.18632/oncotarget.11601 (PMC5323184; doi:10.18632/oncotarget.11601)
Supplement: Supplementary file 1 [file oncotarget-07-65676-s001.pdf]

SHP-2 phosphatase contributes to KRAS-driven intestinal oncogenesis but prevents colitis-associated cancer development

SUPPLEMENTARY TABLE

Supplementary Table S1: Clinicopathological parameters of patients in the series of CRC samples analyzed in Figure 1A

Parameters	Value
Age, median (range) (years)	70 (41-90)
Sex	
Male, no (%)	52 (55%)
Female, no (%)	42 (45%)
Tumor localization	
Left + rectum, no (%)	31 (33%)
Right + transverse, no (%)	63 (67%)
Tumor stage (TNM)	
Adenoma, no (%)	9 (9.5%)
1, no (%)	8 (8.5%)
2, no (%)	30 (32%)
3, no (%)	32 (34%)
4, no (%)	15 (16%)
